# Drug Repurposing for Triple-Negative Breast Cancer

**DOI:** 10.3390/jpm10040200

**Published:** 2020-10-29

**Authors:** Marta Ávalos-Moreno, Araceli López-Tejada, Jose L. Blaya-Cánovas, Francisca E. Cara-Lupiañez, Adrián González-González, Jose A. Lorente, Pedro Sánchez-Rovira, Sergio Granados-Principal

**Affiliations:** 1 GENYO, Centre for Genomics and Oncological Research, Pfizer/University of Granada/Andalusian Regional Government, PTS Granada, Avenida de la Ilustración, 18016 Granada, Spain; martaam97@gmail.com (M.Á.-M.); aracelilopeztejada@gmail.com (A.L.-T.); jose.blaya@genyo.es (J.L.B.-C.); francisca.cara@genyo.es (F.E.C.-L.); adrikangus@gmail.com (A.G.-G.); jose.lorente@genyo.es (J.A.L.); 2 UGC de Oncología Médica, Complejo Hospitalario de Jaén, 23007 Jaén, Spain; oncopsr@yahoo.es; 3 Department of Legal Medicine, School of Medicine—PTS—University of Granada, 18016 Granada, Spain

**Keywords:** triple-negative breast cancer, personalized medicine, computational methods, drug repurposing, clinical trials, cancer stem cells

## Abstract

Triple-negative breast cancer (TNBC) is the most aggressive type of breast cancer which presents a high rate of relapse, metastasis, and mortality. Nowadays, the absence of approved specific targeted therapies to eradicate TNBC remains one of the main challenges in clinical practice. Drug discovery is a long and costly process that can be dramatically improved by drug repurposing, which identifies new uses for existing drugs, both approved and investigational. Drug repositioning benefits from improvements in computational methods related to chemoinformatics, genomics, and systems biology. To the best of our knowledge, we propose a novel and inclusive classification of those approaches whereby drug repurposing can be achieved in silico: structure-based, transcriptional signatures-based, biological networks-based, and data-mining-based drug repositioning. This review specially emphasizes the most relevant research, both at preclinical and clinical settings, aimed at repurposing pre-existing drugs to treat TNBC on the basis of molecular mechanisms and signaling pathways such as androgen receptor, adrenergic receptor, STAT3, nitric oxide synthase, or AXL. Finally, because of the ability and relevance of cancer stem cells (CSCs) to drive tumor aggressiveness and poor clinical outcome, we also focus on those molecules repurposed to specifically target this cell population to tackle recurrence and metastases associated with the progression of TNBC.

## 1. Introduction

Breast cancer is the second most common cancer and the second cause of cancer death among US women, after lung cancer [1]. In 2020, it is estimated that 279,100 new cases will be diagnosed in the United States and more than 42,000 deaths will be a consequence of this type of cancer [2]. It is a heterogeneous disease that has been classified using immunohistochemical techniques to measure the presence of three receptors: estrogen receptor (ER), progesterone receptor (PR), and overexpression of human epidermal growth factor receptor 2 (HER2). Triple-negative breast cancer (TNBC) is characterized by the lack of expression of these receptors and, consequently, there are no approved targeted therapies [3]. Approximately 10% to 20% of new cases of breast cancer would be included in this subtype, which presents poor prognosis with high risk of relapse compared to other breast cancer subtypes [4]. TNBC is the breast cancer subtype with the poorest overall survival (OS) and the highest rates of metastases [5], most commonly in lungs and brain [6]. Furthermore, it is more frequent in women in younger ages and black race, presenting an incidence rate about twice as high compared with white race [1].

Histopathologically, TNBC is a heterogeneous group that mostly presents features of ductal invasive carcinomas, but also metaplastic, medullary, or apocrine characteristics. Based on the gene expression profile, TNBC is divided into four subtypes: basal-like 1 (BL1), basal-like 2 (BL2), luminal androgen receptor (LAR), and mesenchymal (M) [6]. As a result of the variety and the lack of receptors of TNBC, there are not targeted therapies, making it necessary the application of personalized medicine. Whereas TNBC has a higher sensitivity to chemotherapeutics in comparison to other breast cancers, this subtype presents a higher risk of recurrence, which makes the unraveling of new treatments important [5]. Nevertheless, the process of creating and testing a new drug for TNBC is a cost- and time-consuming challenge that requires a huge investment and comprises high failure rates. For this reason, drug repurposing has been considered an increasingly successful approach for developing new therapies [7].

## 2. Current Treatments for TNBC

Besides surgery, nowadays, chemotherapy is the only treatment approved by the Food and Drug Administration (FDA) for non-metastatic TNBC [8], which includes microtubule inhibitors, anthracyclines, alkylating agents, antimetabolites, and platinum (Table 1) [7,9]. The current standard of treatment is based on a combination of anthracyclines and taxane agents [10]. In spite of initial chemosensitivity of tumors and the use of different drug combinations to potentiate treatments, later chemoresistance is frequently developed and it is related to the high presence of cancer stem cells (CSC) [9]. All of these compounds are repurposed drugs as they have been previously approved for diseases other than TNBC [7,11,12].

Additional therapeutic options have been recently approved by the FDA for metastatic TNBC, when patients do not respond to traditional treatments (Table 2) [13]. For instance, olaparib and talazoparib, two PARP (poly[adenosine diphosphate-ribose] polymerase) inhibitors of enzymes were approved for patients harboring germline mutations in *BRCA1/2* [8,13,14,15]. 

Furthermore, the use of patient’s immune system as an approach for cancer treatment, or immunotherapy, has strongly emerged as the fifth pillar of cancer therapy [16]. Immune escape is hallmark of tumor cells that promotes their development and progression, by decreasing immune recognition, for example, through the expression of immune suppressive molecules, or immune checkpoints, like cytotoxic T-lymphocyte-associated antigen-4 (CTLA-4) or programmed cell death-1 and their ligands (PD-1, PD-L1/2)(19–21). Ligand-receptor binding inhibits T-lymphocytes activity through their exhaustion. Physiologically, these molecules are checkpoint regulators of strength and last of LT-mediated immune response [16]. Interaction of PD-1/PD-L1 represents a mechanism of resistance to adaptative immune system by tumor cells in response to the endogenous antitumor response [16]. Nowadays, several checkpoint inhibitors (CPIs) (antibodies anti-CTLA-4, anti-PD-1, and anti-PD-L1) are under clinical use in cancer. In TNBC, combination of CPIs with targeted therapies and/or chemotherapy have been shown to be more effective than monotherapy, which showed a modest effectivity and durability [17]. Recently, atezolizumab, an inhibitor that targets PD-L1, has been approved in combination with paclitaxel for the treatment of patients with previously untreated metastatic TNBC (IMpassion130 study, NCT02425891) [18,19]. Despite of the great expectative on this new and expensive therapy, a small percentage of patients respond to it [16] because of several reasons such as the low tumor infiltration of lymphocytes (TILs, tumor infiltrating lymphocytes), presence of which is associated with a higher survival and good prognosis in early stage TNBC patients [17], low expression of PD-L1 on tumor cells, or the expression of other inhibitor molecules of immune system (IDO, CD73, TIGIT, or VISTA) [20].

Lastly, antibody-drug conjugates (ADC) represent a big potential to improve cancer treatment as they allow to target toxic drugs directly into cancer cells by using specific receptors. Sacituzumab govitecan is the newest therapeutic option available only after the failure of at least two other treatments [13]. This FDA-approved drug is an anti-trophoblast cell-surface antigen 2 (Trop-2) antibody conjugated with SN-38, a DNA damaging agent [21]. 

## 3. Drug Repurposing

**Figure 1:**
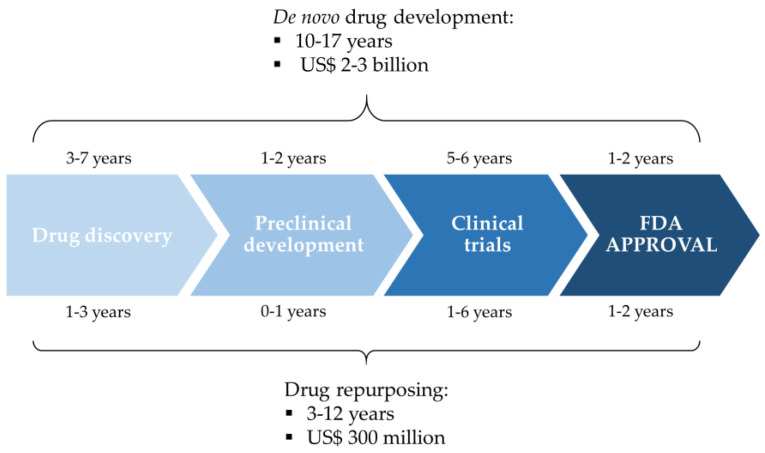
Comparison between de novo drug development and drug repurposing. Adapted from Ashburn and Thor [22].

The discovery and development of a new drug is a time-consuming process which requires great investments, being estimated to take between 10 and 17 years and a cost of US$2–3 billion [22,23]. Moreover, it comprises high failure rates in clinical trials, where almost 90% of the drugs are rejected because of unexpected properties [7]. Drug repurposing (also known as drug repositioning or drug reprofiling) is a strategy for identifying new uses for existing drugs, both approved and under investigation (Figure 1). This relatively new strategy allows to significantly shorten the time and reduce the costs of drug development, especially in the case of FDA-approved repurposed drugs, which would likely go through accelerated clinical trials owing to their previous safety and toxicological clinical studies [24]. It has been estimated that repurposing a drug would cost, on average, US$300 million [23]. Several methodologies can be considered for drug repurposing, from non-computational approaches including high-throughput screening [25] and methods based on experimental findings and previous literature, e.g., target-based, to computational strategies. Indeed, drug repurposing process can be highly improved via computational methods related to chemoinformatics, genomics, and systems biology. These methods allow to select, prior to in vitro experiments, drug candidates for repositioning in a rational manner [24,26,27].

### 3.1. Common Computational Approaches for Drug Repurposing 

**Figure 2:**
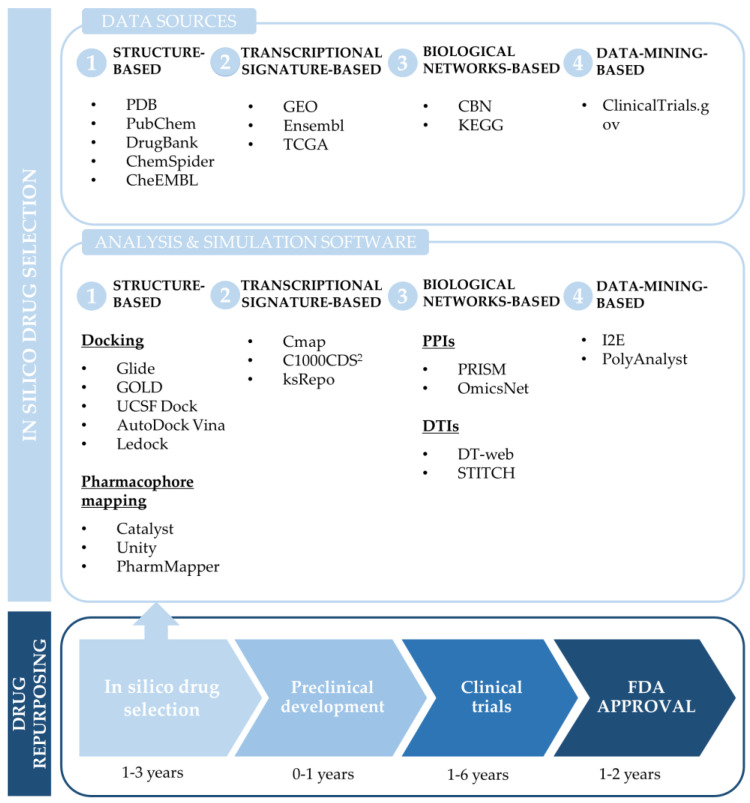
Diagram of the main computational approaches and software for drug repurposing.

There are many different computational approaches for drug repurposing based on different types of data, including drug and target structures, drug–target interactions, or transcriptomes. Accordingly, several classifications have been suggested [24,28,29]. To date, it has not been determined which approach would be the best option for in silico drug repositioning, and no standardized method has been adopted. Hence, analyzing the retrieved literature, it was considered of interest reviewing and summarizing the most accessible, commonly used approaches (Figure 2), so as to provide a fuller view of the current strategies and the possibilities that in silico analysis has to offer. Thus, these various computational approaches have been categorized in: (1) structure-based, (2) transcriptional signatures-based, (3) biological networks-based, and (4) data-mining-based drug repurposing.

#### 3.1.1. Structure-Based Drug Repurposing

Structure-based methods, which rely on both drug and receptor structure, are mainly based on virtual high-throughput screening (VHTS) of small chemical compounds from different databases such as PubChem (https://pubchem.ncbi.nlm.nih.gov/), DrugBank (www.drugbank.ca/), ChemSpider (www.chemspider.com/) or CheEMBL (www.ebi.ac.uk/chembl/). It allows the user to find, in silico, multiple drugs that will potentially interact with the target’s binding site [24]. The 3D structure of the target, which is usually a protein, can be found in the Protein Data Bank (PDB, www.rcsb.org/). VHTS comprises a computational modelling technique known as molecular docking, which enables to predict ligand-receptor biding affinity via different scoring functions. There are several molecular docking programs, such as Glide (www.schrodinger.com/glide), GOLD (www.ccdc.cam.ac.uk/solutions/csd-discovery/components/gold/), UCSF DOCK (http://dock.compbio.ucsf.edu/), AutoDock Vina (http://vina.scripps.edu/), or Ledock software [30]. VHTS can also be inversely approached by finding a variety of biological targets that may have affinity for a particular ligand. Apart from molecular docking, the user can also perform pharmacophore mapping, which consists of searching of ligands that can be matched to a pharmacophore, i.e., a set of molecular features such as hydrogen bonds, hydrophobic groups, or chemical substructures, that enable the recognition of a ligand by a receptor and their biological activity. Pharmacophore features can be derived from protein-binding site or protein–ligand complexes structures, and software packages such as Catalyst (www.3dsbiovia.com/), Unity (Tripos, www.tripos.com), or PharmMapper can be used for pharmacophore searching [24,26]. Structure-based methods also encompass ligand/receptor profiling, based on a guilt-by-association principle. Ligand profiling consists of finding compounds that are chemically similar to a given drug, and consequently may have similar functional and biological properties. Likewise, receptor profiling consists of finding proteins that have similar binding sites to a particular receptor, therefore being likely to bind with the same ligands [24,26]. 

#### 3.1.2. Transcriptional Signature-Based Drug Repurposing

Transcriptional signatures related to a disease or transcriptional responses associated to a specific treatment can be used for drug repurposing. Potential drug candidates can be identified via negative correlation between the gene expression profile from a disease and the transcriptional signature induced by a small compound, with the aim of finding a drug that would reverse the disease state toward the normal one. Similarly, positive correlation can be used to identify small compounds that have similar transcriptional signatures to a genetically or chemically induced perturbation, so as to induce a similar gene expression [31]. Signature-based drug repurposing is also known as connectivity mapping, a concept first introduced with the creation of the Connectivity Map (CMap) database [32,33], which comprises a genome-wide dataset of transcriptional expression responses of human cell lines to perturbagens, e.g., chemical treatments or genetic perturbations [34]. Transcriptional data can be found in different public databases such as Gene Expression Omnibus (GEO; www.ncbi.nlm.nih.gov/geo/), Ensembl (www.ensembl.org/), or The Cancer Genome Atlas (TCGA; https://portal.gdc.cancer.gov/), and several tools are available for analyzing and comparing drug and disease transcriptional profiles. Examples of tools for signature-based repurposing are CMap (https://clue.io/), L1000CDS^2^ (http://amp.pharm.mssm.edu/L1000CDS2/), and ksRepo free source [24].

#### 3.1.3. Network-Based Drug Repurposing

Biological networks are data representations used to model biological interactions of any kind, where nodes represent various biological components, such as genes or proteins, and whereas edges represent the associations between them [28]. Network-based drug repositioning methods help inferring unknown disease-associated signaling pathways and therefore new therapeutic targets. There are different biological networks depending on the main source of biological data. Some interesting examples are protein–protein interaction (PPI) networks and drug–target interaction (DTI) networks. In PPI networks, nodes represent proteins. Most proteins are associated with other proteins, but only a limited number of them interact with multiple others. PPI networks allow to identify the most highly connected central proteins, generally known as hubs or hub proteins [35]. Alterations of hubs may affect the structure of the biological network, leading to dysfunction and disease [36]. Accordingly, PPI networking methods help predicting new disease-related targets for drug repurposing. PPI analysis can be performed with PRISM (Protein Interactions by Structural Matching; http://gordion.hpc.eng.ku.edu.tr/prism) server [36], or OmicsNet (https://omicsnet.ca/). Regarding DTIs, they are considered bipartite networks, where nodes represent both drugs and targets. There are several tools for predicting potential DTIs, such as DT-web (https://alpha.dmi.unict.it/dtweb/) or STITCH (http://stitch.embl.de/). Moreover, systems biology combines different network models with quantitative mathematical network models to infer the dynamics of biological systems, providing a more complete perspective for drug repurposing [24]. Complex biological networks can be found in the Causal Biological Networks (CBN, http://causalbionet.com/) database, and complex biological pathways can be found in KEGG database (www.kegg.jp/). 

#### 3.1.4. Data-Mining-Based Drug Repurposing

All the previously described methods are based on drug–target interactions. However, meta-analysis of data from clinical trials is another interesting approach for drug repurposing. Su et al. [37] described a novel method for drug repositioning using ClinicalTrials.gov (https://clinicaltrials.gov/) public database and two text mining tools, I2E (Linguamatics) and PolyAnalyst (Megaputer). It consists of, first, the extraction of Serious Adverse Event (SAE) data to identify drugs with fewer SAEs on the test arm than on the control arm and, second, the ranking of said drugs. Therefore, it allows to discover potential drug candidates for diseases different from those in the testing conditions.

## 4. Drug Repurposing for TNBC

**Figure 3:**
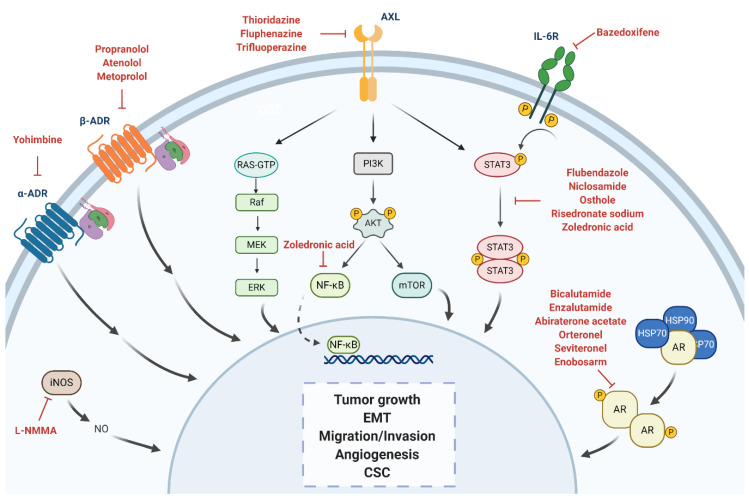
Overview of the different pathways investigated by drug repurposing. Repurposed inhibitors under investigation are shown in red. Created with BioRender.com.

The urgent necessity to find effective molecularly targeted treatments for TNBC has been translated into efforts by the research community to characterize and divide it into different subtypes with a more approachable profile. One of the first transcriptomic-based breast cancer classifications was performed by Perou et al., using cDNA microarrays and hierarchical clustering analysis to distinguish variations in gene expression patterns [38]. It gave a different approach to the commonly immunohistochemical characterization of breast cancers. Afterwards, several studies conducted similar genome-wide analyses [39,40,41], up until 2009 when Bernard et al. developed a qRT-PCR-based assay using only fifty genes (PAM50) to classify tumors into four intrinsic subtypes of breast cancer: luminal A, luminal B, HER2-enriched, and basal-like [42]. In 2007, Kreike et al. performed the first gene-expression-based classification of TNBC. After gene profiling, they identified all triple-negative breast tumors as basal-like, and classified them in five different subgroups [43]. In opposition, Prat et al. proved that basal-like cancers were not interchangeable with TNBCs [44], similarly to the findings of the study conducted by Lehman et al. in 2014 [45]. While the majority of TNBCs are basal-like, and vice versa, they should not be considered synonymous. These studies highlighted the necessity to further classify TNBC in well-defined subtypes in order to successfully develop personalized therapies. The first transcriptomic-based TNBC classification which differentiated between basal-like and non-basal like TNBC subtypes was performed by Lehman et al. in 2011. They identified six TNBC subtypes with representative gene expression signatures and signaling pathways, including two basal-like (BL1, BL2), an immunomodulatory (IM), a mesenchymal (M), a mesenchymal stem-like (MSL), and a luminal androgen receptor (LAR) subtype [46]. A web-based tool (TNBCtype) was also developed for the classification of TNBC samples into the six mentioned subtypes [47]. Later in 2016, Lehman et al. refined their own classification algorithm and developed a new one (TNBCtype-4), which scaled down the number of subtypes to four: BL1, BL2, M, and LAR [48]. While several other TNBC classifications followed different approaches and described varying number of subtypes, they all broadly concurred in those four main subgroups [49,50,51]. Recently, based on both Lehman et al. and Ring et al. algorithms [48,52], Espinosa et al. identified various TNBC cell lines whose signatures remained stable between cell lines and xenografts for each of the four subtypes: HCC2157 for BL1 subtype; HCC70, SUM149PT and HCC1806 for BL2 subtype; BT-549 for M subtype; and MDA-MB-453 for LAR subtype [53]. Thus, those cell lines, representative of each subtype, should be considered for in vitro studies on the effectiveness of targeted therapies in all different subtypes. Among the previously mentioned TNBC subtypes, the dependency on androgen receptor (AR) signaling of the LAR subtype provides a feasible target for directed therapies, which makes it an excellent candidate for drug repurposing. Whereas patients with AR-dependent TNBCs, which have a better prognosis than those with other TNBC subtypes [54], would benefit from AR inhibition therapy, it has been suggested that this may also be beneficial for non-LAR patients with relatively lower AR expression [50,55,56]. However, not all TNBCs express AR, so a quadruple negative breast cancer subtype has also been addressed [57,58]. This subtype would not benefit from AR antagonist repurposing treatments, and so forth different molecular pathways would need to be targeted. Accordingly, we offer an insight on the main repurposed therapies which are currently being investigated for the treatment of TNBC based on their molecular targets, including both AR-directed and non-AR-directed therapies, as shown in Figure 3. We have also summarized drugs in preclinical phase for TNBC in Table 3 and those under clinical trials in Table 4.

### 4.1. Androgen Receptor

LAR subtype is highly enriched in hormonally regulated pathways, despite being negative for both ER and PR. All ER, PR, and AR belong to the nuclear steroid hormone receptor family, and it has been proposed that AR overexpression may replace ER signaling, resulting in similar functional effects. In fact, both epidemiological and preclinical studies suggest that the androgenic signaling pathways may be linked to the development of breast cancer [50,51,54]. AR plays a central role in regulating gene expression, is mainly located in the cytoplasm, and it can be found complexed with heat shock proteins, HSP70 and HSP90, in order to maintain its inactive conformation. Upon binding of androgens, the receptor dissociates from HSPs and homodimerizes, enabling nuclear translocation. Once in the nucleus, AR binds to the promoter of target genes and induces the recruitment of coactivators and other transcription factors, therefore inducing transcriptional activation [54,95]. In TNBC, it has been suggested that AR activation alters the tumor microenvironment, hence suppressing the antitumor response and upregulating the secretion of the epidermal growth factor receptor (EGFR) ligand amphiregulin (AREG), both stimulating tumor growth and progression. AR activation has also been linked to metastasis via promotion of epithelial-to-mesenchymal transition (EMT), survival of anchorage-independent cell population, and maintenance of a CSC-like population [56,58]. However, the mechanisms by which AR-associated pathways may influence TNBC development and progression still remain unclear and are currently under research. Considering the crucial role that AR may play in AR-positive TNBC, different AR-targeted agents first intended for the treatment of metastatic castration-resistant prostate cancer (mCRPC) are being repurposed and tested in clinical trials on TNBC patients. It includes several FDA-approved drugs, such as bicalutamide, enzalutamide, or abiraterone acetate, as well as experimental drugs such as orteronel or seviteronel [88,96,97]. In fact, enzalutamide has proved to prolong survival in men with mCRPC after developing drug resistance to chemotherapy [98]. Therefore, they might represent an alternative treatment to avoid resistance in TNBC. Additionally, selective AR modulators or SARMs (e.g., enobosarm), investigational drugs first intended to be used as an alternative to testosterone therapies for male hypogonadism as well as related conditions such as muscle dystrophy, sarcopenia, or osteoporosis, are also currently being tested in clinical trials for both prostate cancer and TNBC [95,99,100]. 

Bicalutamide. It was the first drug to be repurposed in clinical trials as a potential treatment for AR-positive TNBC. Bicalutamide is a first-generation, non-steroidal antiandrogen developed for prostate cancer. It acts as a competitive inhibitor that directly binds to AR, stabilizing its association with HSPs. Whereas it maintains the receptor in an inactivated conformation, it does not prevent nuclear translocation and binding to DNA, which entails possible partial agonistic activity [58,101]. In vitro studies showed that bicalutamide significantly reduced cellular proliferation and colony formation, and induced cell apoptosis in MDA-MB-453 and MDA-MB-231 breast cancer cells. Reduction of tumorigenicity was associated with the inhibition of Wnt/β-catenin signaling pathway through downregulation of c-Myc transcripts. Moreover, assays with xenografts tumors of MDA-MB-453 and MDA-MB-231 cells further demonstrated that bicalutamide decreased cellular viability and induced apoptosis in vivo [82]. A single-arm, nonrandomized, phase II clinical trial with bicalutamide was performed in AR-positive TNBC (NCT00468715). The criteria to define AR positivity was an AR expression higher than 10% by immunohistochemistry (IHC). Among all AR-positive patients (*n* = 51), 26 were treated with bicalutamide. The clinical benefit rate (CBR), defined as the total number of patients who showed a complete response, partial response, or stable disease at 6 months, was 19%, and the median progression-free survival (PFS) was 12 weeks. The drug had grade 1–3 adverse events (AEs), such as fatigue, limb edema, or hot flashes, indicating a moderate toxicity. This study suggested the potential of AR blockade in AR-positive metastatic TNBC [81]. Other clinical trials are currently under development, including a phase II (NCT02605486) and a phase III (NCT03055312) trial.

Enzalutamide. It is a second-generation, non-steroidal antiandrogen developed for prostate cancer, with higher binding affinity than bicalutamide. Upon binding to AR, enzalutamide blocks nuclear translocation, recruitment of AR cofactors, and transcriptional activation which, oppositely to bicalutamide, results in a lack of agonistic activity [54,55,58]. Different in vitro studies demonstrated that enzalutamide reduced cell proliferation, migration, and invasion and increased apoptosis [55,56,84], and it was correlated with decreased AREG mRNA expression in SUM159 cells after treatment with enzalutamide [56]. In vivo studies showed that enzalutamide inhibited tumor viability in TNBC xenografts by inducing cell apoptosis [56,84]. A single-arm, non-randomized phase II clinical trial evaluated the efficacy of enzalutamide in advanced AR-positive TNBC (NCT01889238). In this study, AR positivity was defined as AR expression higher than 0% by IHC (intent-to-treat population, ITT) or higher than 10% by IHC (evaluable subgroup). The ITT population (*n* = 118) and the evaluable subgroup (*n* = 78) showed a CBR at 16 weeks of 25 and 33%, respectively. Median PFS was 2.9 months in the ITT group and 3.3 in the evaluable group. Median OS was 12.7 and 17.6 in ITT and evaluable subgroup, respectively. The only treatment-related AE with grade 3 or higher was fatigue, meaning enzalutamide was well tolerated by AR-positive TNBC patients. This study supported further study of enzalutamide [83]. Moreover, other clinical studies are currently investigating the use of enzalutamide as an adjuvant in treating patients with AR-positive TNBC, including a phase II trial (NCT02689427) for enzalutamide in combination with paclitaxel and a phase Ib/II trial for enzalutamide in combination with taselisib (NCT02457910).

Abiraterone acetate. It was the first androgen-production inhibitor developed for the treatment of prostate cancer. It is a steroidal, non-selective inhibitor of 17α-hydroxylase/17,20-lyase (CYP17), a central, rate-limiting enzyme which plays a critical role in the androgen biosynthesis pathway [54,58,102]. The efficacy of abiraterone acetate was investigated in a phase II clinical trial in combination with prednisone in metastatic or locally advanced AR-positive TNBC patients (NCT01842321). AR positivity was defined as AR expression greater than 10% by IHC. Evaluable patients (*n* = 30) showed a CBR at 6 months of 20%, and the median PFS was 2.8 months. The most common treatment-related AEs were hypertension, fatigue, nausea, and hypokalemia, all grade 1–2 [85]. After this clinical trial, both in vitro and in vivo studies were performed to assess whether combining abiraterone acetate with a Chk1 inhibitor would enhance its efficacy. They showed that combination treatment with the inhibitor GDC-0575 had an additive effect on both MDA-MB-453 and SUM185PE cell lines in reducing cell proliferation. Whereas abiraterone acetate alone had a weak effect inducing apoptosis, Chk1 inhibitors doubled the effect, achieving statistical significance in MDA-MB-453 cells. Interestingly, a xenograft model with MDA-MB-453 cells injected orthotopically in the mammary gland ducts of NSG mice showed that abiraterone alone reduced tumor growth, and combination with GDC-0575 enhanced this effect [86].

Orteronel (TAK-700). It is a non-steroidal, selective, second-generation CYP17 inhibitor. Whereas clinical trials for the treatment of prostate cancer with orteronel were terminated in phase III because of a lack of significant effect on OS [54,58,103], it is currently being investigated in a phase II clinical study of women with AR-positive metastatic TNBC (NCT01990209).

Seviteronel (VT-464). It is another non-steroidal, selective, second-generation CYP17 inhibitor which, in contrast to orteronel, also inhibits AR activation [54,58]. It was demonstrated that seviteronel inhibited cellular growth and tumor volume in MDA-MB-453 cells and patient-derived xenografts (PDX), respectively [88,89]. Moreover, Michmerhuizen et al. proved that the AR inhibition with seviteronel induced radiosensitization, both in vitro and in vivo, whereas enzalutamide did not [104]. A phase I/II clinical study is investigating the activity of seviteronel in women with AR-positive TNBC (NCT02580448). Out of 16 patients with AR-positive TNBC, 6 were evaluable. Two patients (33%) had a 16-week CBR. The most common AEs were fatigue, nausea, and decreased appetite, all grade 1–2 [87]. A second phase II clinical trial is also currently investigating the effects of seviteronel in AR-positive TNBC patients (NCT02130700).

Enobosarm (MK-2866, ostarine, GTx-024). It is a non-steroidal SARM that achieves a tissue-selective modulation of AR action, hence minimizing the undesirable side-effects caused by antiandrogens [105]. In vitro studies showed that enobosarm inhibited cellular proliferation of MDA-MB-231 cells transiently expressing AR. Moreover, tumor growth was completely inhibited by enobosarm in a nude mice xenograft model with MDA-MB-231-AR cells [106]. There was a phase II clinical trial for enobosarm in AR-positive TNBC (NCT02368691), but it was terminated because of lack of efficacy.

### 4.2. Adrenergic Receptor

Adrenergic receptors (ADR), which can be classified as α or β receptors, belong to the G protein-coupled receptor (GPCR) superfamily. The activation of ADR, stimulated through the catecholamines, epinephrine and norepinephrine, derives in several stress response signaling pathways key in maintaining physiological homeostasis [107]. However, there is an increasing evidence that altered ADR stimulation may play a significant role in breast cancer progression, promoting cell proliferation, metastasis, tumor invasion, and angiogenesis [68,108,109]. Accordingly, it has been addressed that ADR-directed therapies, widely used for the treatment of hypertension and other pathologies, could be repurposed for TNBC. Several preclinical studies have investigated the effects of both α- and β-ADR antagonists in TNBC [61,64,66,67,110,111], and retrospective epidemiological studies have explored whether TNBC cancer patients under treatment with beta-blockers for hypertension had a significant better outcome that non-treated patients [63,68,108,112]. 

#### 4.2.1. α-Adrenergic Receptor

α-adrenergic receptors can be subclassified as α1 (α1a, α1b, α1c) and α2 (α2a, α2b, α2c). Their ligands activate GPCRs and initiate a signaling cascade that, in the case of α1 receptors, increases intracellular calcium levels and is involved in blood pressure regulation, whereas α2 receptors signaling cascade decreases intracellular cyclic AMP (cAMP) levels and regulates neurotransmitters release [107]. Interestingly, activation of α-ADR has been associated with both tumor growth and chemoresistance in TNBC cell lines. Vazquez et al. showed that both epinephrine and norepinephrine, the natural ADR agonists, as well as clonidine, a synthetic α(2)-ADR agonist used in the treatment of hypertension [113], promoted cell proliferation in MDA-MB-231 cells [110]. Similarly, Bruzzone et al. demonstrated that clonidine increased tumor growth, whereas α(2)-ADR antagonist **α-**yohimbine reversed clonidine stimulation in breast cancer [114].

α-yohimbine (rauwolscine). It is an alkaloid and α(2)-ADR antagonist used as a mydriatic and in the treatment of impotence [115]. Piñero et al. found that yohimbine diminished tumor growth in vitro, and it was associated with inhibition of ERK1/2 phosphorylation in vivo [61]. It was also proved that **α-**yohimbine could reverse tumor growth after stimulation with clonidine in vivo [59]. Additionally, Flint et al. demonstrated that MDA-MB-231 cells developed resistance to paclitaxel when treated in combination with catecholamines and/or cortisol [60]. In the light of these results, we suggest the investigation of α-ADR antagonists for the treatment of TNBC and prevention of drug resistance.

#### 4.2.2. β-Adrenergic Receptor

β-adrenergic receptors can also be subclassified as β1, β2, and β3. Activation of β1- and β2-ADR increases intracellular cAMP levels, as opposed to α2-ADR, regulating the sympathetic nervous system’s stress response in several different tissues [107]. The signaling cascade induced by higher cAMP levels includes two main pathways. First, cAMP activation of protein kinase A (PKA) induces phosphorylation of several transcription factors, such as GATA family, and β-ADR kinase (BARK). The latter inhibits β-ADR signaling and activates Src kinase, leading to the activation of different transcription factors, including STAT3, and several kinases like focal adhesion kinase (FAK). Conversely, cAMP also leads to Rap1A activation, which induces B-Raf/mitogen-activated protein kinase (MAPK) signaling pathway and activation of multiple genes with effects on several cellular events [116]. It has been addressed that, in breast cancer, β-ADR signaling in β-ADR-expressing tumor cells activates metastatic-associated genes involved in inflammation, angiogenesis, and EMT processes, whereas it downregulates the expression of antitumoral response genes. Moreover, activation of β-ADR pathway in tumor stromal cells and tumor-associated macrophages seem to promote tumor growth and metastasis [109,116]. Several in vitro studies with different TNBC cell lines showed that β-ADR agonists stimulated cell migration, whereas β-ADR antagonists, such as atenolol and ICI118551, reverted this process [66,67,111]. Moreover, it was also demonstrated that β-blockers propranolol and ICI118551 decreased cell proliferation in TNBC, arresting the cell cycle and inducing cell apoptosis [62]. Oppositely, Slotkin et al. showed that treatment with β-ADR agonist isoproterenol lowered DNA synthesis and decreased cell proliferation, and that these effects were reverted by propranolol [64]. Similarly, in an experimental mouse model of breast cancer, β-ADR agonists isoprenaline and salbutamol inhibited breast cancer cell proliferation and tumor growth [61]. There seems to be conflicting results in the role of β-ADR signaling in breast cancer, indicating that it might be dependent on the cancer subtype. Accordingly, different retrospective observational cohort studies have been developed to further study the effects of different non selective β1/β2-blockers (propranolol, timolol) and selective β1-blockers (atenolol, bisoprolol, metoprolol) in breast cancer, more precisely in TNBC, so as to determine their effects in the cancer biology of each subtype [63,68,108,112]. The first observational study was performed by Powe et al. [108], in which breast cancer patients were divided into three subgroups: non-hypertensive control group (*n* = 374), hypertensive patients treated, prior to cancer diagnosis, either with β-blockers (*n* = 43) or with other antihypertensives (*n* = 49). Most β-blocker users had received selective blockers (25 with atenolol, 7 bisoprolol), but several had received non-selective ones (7 propranolol, 4 timolol). β-blocker users group suggested a significant lower risk of metastasis development, tumor recurrence, and breast cancer mortality. However, differences in β-ADR antagonists used by patients, and the lack of information in their cancer subtype made it necessary to perform further studies to assess the efficacy of non-selective β1/β2-blockers versus selective β1-blockers in TNBC.

Non-selective β1/β2-blockers (propranolol). Different studies showed that propranolol inhibited cell proliferation, arrested the cell cycle at G0/G1 and S, and induced cell apoptosis in vitro, and inhibited tumor growth in vivo [61,62,65]. Moreover, the anti-tumorigenic effects of this β-blocker were associated with a decrease in phosphorylation levels of ERK1/2 and the expression levels of cyclooxygenase 2 (COX-2) [62]. Interestingly, Pasquier et al. reported that, whereas combination of propranolol with chemotherapeutic drug paclitaxel seemed to have no additive effects in cellular cytotoxic effects in vitro, propranolol increased the anti-tumor efficacy of paclitaxel in an orthotopic xenograft model of TNBC, significantly increasing the median survival [65]. Barron et al. performed a study on women treated with propranolol for hypertension (*n* = 70) in the year before breast cancer diagnosis, in comparison with matching (1:2) non-users (*n* = 4738), and suggested that the use of propranolol was significantly associated with less advanced disease at diagnosis and decreased risk of metastasis and mortality [63]. However, like Ganz et al. pointed out, the limited size of the β-blocker users’ group may be insufficient to prove propranolol benefits in breast cancer [117]. Moreover, the patient population was not subclassified based on cancer subtype or receptor status, so no conclusions can be drawn for TNBC subtype.

Selective β1-blockers (atenolol, metoprolol). In vitro studies demonstrated that atenolol inhibited cell proliferation in MDA-MB-435 cells [69], and enhanced metformin activity in vivo by reducing angiogenesis and metastasis [70]. In the same study mentioned above, Barron et al. also evaluated breast cancer patients treated with selective β1-blocker atenolol (*n* = 525) in the year before cancer diagnosis. However, they found no significant difference in between atenolol users and matched non-users in tumor incidence, risk of metastasis and mortality rates. These results indicated that the effects of propranolol in breast cancer were mediated by β2-ADR [63]. Melhem-Bertrandt et al. performed another retrospective study comparing breast cancer patients treated with β-blockers (*n* = 102), who received neoadjuvant chemotherapy, with non β-blockers users (*n* = 1311), as well as TNBC patients taking β-blockers (*n* = 29) compared to non-users (*n* = 348) [68]. The most commonly prescribed β-blockers were selective β1-blockers, first metoprolol (42%) followed by atenolol (37%). Interestingly, after age, race, stage, and receptor status adjustment, among some other parameters, users of β-blockers proved to have significantly lower recurrence but no significant OS among both breast cancer and TNBC patients, which seemed to contradict the findings of Barron et al. However, a subset analysis demonstrated that the subgroup of ER-positive breast cancer patients had no significant differences in tumor recurrence. Consequently, these results suggested that, whereas patients with any breast cancer subtype could benefit from a treatment with non-selective β-blockers via β2-ADR antagonism, only TNBC patients could benefit from a treatment with non-selective β-ADR inhibitors. Nevertheless, it has to be noted that not statistically significant results in the ER-positive subgroup may have been due to the relatively short follow-up time in the study of Melhem-Bertrandt et al. Additionally, in a retrospective study on TNBC patients taking β-blockers (*n* = 74), compared to non-users (*n* = 726), Botteri et al. also demonstrated that a treatment with β-blockers was associated with a decreased risk of recurrence, metastasis, and mortality, supporting previous findings [112]. Nevertheless, new prospective studies will be required to clarify whether the efficacy of β-blockers depends on breast cancer subtype and/or receptor status.

### 4.3. STAT3

Signal transducer and activator of transcription 3 (STAT3) is a tumor marker for early diagnosis and the activation of its pathway is related to breast cancer aggressiveness, as it plays an important role in progression, proliferation, apoptosis, metastasis, and chemoresistance [118]. The activation of this pathway involves several cytokines such as, interleukin 6 (IL-6) and interleukin 10 (IL-10), and growth factors, including epidermal growth factor (EGF), fibroblast growth factor (FGF), and insulin-like growth factor (IGF), which bind their receptors and activate Janus kinases (JAKs). JAKs phosphorylate themselves in a tyrosine domain included in their cytoplasmic fractions and they subsequently activate STAT3 via tyrosine phosphorylation. Once STAT homodimers are produced, they are translocated to the nucleus in order to create a complex with coactivators (e.g., p68) and ending up into the activation of transcription [118]. The upregulation of IL-6/STAT3/ROS can lead to the transcription of genes involved in breast cancer progression, as well as an augmentation in inflammation and generation of breast cancer stem cells (BCSCs). Furthermore, the activation of JAK2/STAT3 favors proliferation and motility of breast cancer cells by different mechanisms, including the suppression of apoptosis by upregulation of cyclin D-1, c-Myc, and Bcl-2, and promotion of EMT. Finally, resistance to several drugs like paclitaxel may be a consequence of this pathway. Because of its complexity and wide regulation of breast cancer cells, STAT3 is an interesting target candidate to treat in TNBC. As a matter of fact, several compounds that inhibit different mechanisms are being investigated. We will highlight some of them: bazedoxifene, flubendazole, niclosamide, osthole, and zoledronic acid [118].

Bazedoxifene. It is a selective ER modulator approved in 2013 by the FDA to treat and prevent osteoporosis in postmenopausal women [71]. Using a structure-based study for repurposing drugs, bazedoxifene was discovered as a novel inhibitor of IL-6 receptor by blocking signals of glycoprotein 130 [119]. Hence, in TNBC, its mechanism involves the upstreaming disruption of STAT3 pathway as ER is not expressed. Studies in in vitro and in vivo models of TNBC confirmed the decrease of cell viability, migration, colony formation, and increase of apoptosis. Furthermore, when this compound was administered in combination with paclitaxel, a synergistic effect as well as an improvement of sensitivity to paclitaxel was found, probably because of the inhibition of the resistance effect induced by IL-6 [71,72]. Those doses were administered in safety ranges that are registered in other indication trials of bazedoxifene. Subsequently, safe effects can be assured in endometrial, ovarian, and breast tissues, but it would be necessary to study possible secondary effects in other tissues that express ER [72]. Considering the association between STAT3 and EMT, their interplay in CSCs, and the in vitro effects of bazedoxifene, we suggest that this compound could act as an inhibitor of tumor-initiating cells, although this hypothesis must be further investigated.

Flubendazole. It is an FDA-approved anthelmintic agent to treat intestinal parasites whose mechanism of action is the disruption of tubulin polymerization. For this reason, it was considered as a repurposed candidate to treat breast cancer [120]. Even though flubendazole causes cell cycle arrest at G2/M phase and, consequently, inhibits cell proliferation in vitro and tumor growth in vivo at clinical doses, it also presents additional properties. As an STAT3 inhibitor, it also causes a reduction of CD44^high^/CD24^low^ CSC population, mammosphere-forming ability, and the expression of stemness genes [73]. This fact is a positive characteristic as CSCs might have an essential role in metastasis and aggressiveness of TNBC [120]. Furthermore, in some studies flubendazole is shown to increase cytotoxicity activity of fluorouracil and doxorubicin, meaning it could reduce tumor chemoresistance [73].

Niclosamide. It is a FDA-approved anthelmintic agent to treat tapeworms, which is known to inhibit cell growth in vitro and tumor growth in vivo in TNBC studies [74]. Niclosamide was identified as an inhibitor of BCSCs owing to a high-throughput drug screening [76]. It reverses EMT and inhibits the stem-like phenotype in cancer cells suggesting that it may reverse cisplatin resistance [74]. Furthermore, Lu et al. proved that niclosamide is a radiosensitizer both in vitro and in vivo models of TNBC as it reversed radioresistance generated by activation of STAT and Bcl-2 and reduction of reactive oxygen species (ROS) [75].

Osthole (7-methoxy-8-isopentenoxycoumarin). It is a coumarin-derivative extract isolated from *C. monnieri* that presents interesting properties, such as anti-inflammatory and vasorelaxant [121]. Osthole has successful results in vivo treating osteoporosis as it stimulates osteoblast proliferation and differentiation and bone formation [77]. It also possesses anti-tumoral characteristics and, hence it can be a candidate for repositioning in TNBC. Dai et al. elucidated that osthole inhibits STAT3 phosphorylation, induced by IL-6, in a dose-dependent manner by avoiding the translocation of STAT3 to the nucleus, what causes cell cycle arrest and induction of apoptosis in TNBC cell lines. Moreover, in vivo assays with osthole confirmed the suppression of STAT3 phosphorylation as well as reduction of tumor growth in TNBC xenograft mice [78]. 

Risedronate sodium and zoledronic acid. They are two oral bisphosphonates to treat osteoporosis that were found to be possible candidates as STAT3 inhibitors by a comparative docking study in silico. Svranthi et al. also proved their toxicity in TNBC cells in vitro [79]. Furthermore, zoledronic acid has been largely analyzed for TNBC. Schech et al. proved that it inhibited cell viability, induced cell cycle arrest, reduced proliferative capacity, inhibited self-renewal capability, and decreased the expression of EMT markers (N-cadherin, Twist, and Snail). Mechanistically, they discovered that zoledronic acid inhibited phosphorylation of RelA, an active subunit of nuclear factor κB (NF-κB). Consequently, direct inactivation of NF-κB induced the loss of EMT transcription factor gene expression [91]. In vivo studies in mice also support the antitumor potential of zoledronic acid in combination with doxorubicin [92]. In a randomized phase II clinical trial (UMIN000003261), the combination of zoledronic acid and neoadjuvant chemotherapy was evaluated in TNBC patients. The pathologic complete response rate (pCR) was ameliorated in the combination group (35.3%) (*n* = 17) compared to patients treated with chemotherapy alone (11.8%) (*n* = 17). Such an improvement of pCR rate was translated into a higher disease-free survival in the combination group (70.6%) versus the chemotherapy group (94.1%) [90]. In contrast, a phase II clinical trial studying the application of pre-operative zoledronate prematurely ended because of a low accrual rate (NCT02347163). Further trials to assess the anti-tumor activity of zoledronic acid are currently ongoing in combination with atorvastatin and neoadjuvant standard chemotherapy (NCT03358017), as well as to evaluate the potential of zoledronic acid as an adjuvant therapy (NCT02595138, NCT04045522). 

### 4.4. Nitric Oxide Synthase

Nitric oxide (NO) is a small molecule that is involved in several functions in the organism. It can be synthesized by three isoforms of nitric oxide synthase (NOS): neuronal (NOS1/nNOS), inducible (NOS2/iNOS), and endothelial (NOS3/eNOS). NO has a short half-life and interacts with different targets, which produces nitrites, nitrates, S-nitrosothiols, and nitrosamines, these being compounds that induce DNA damage and, therefore, gene mutations [122]. Glynn et al. proved that an increased expression of iNOS in ER^–^ breast cancer is correlated with poor survival of patients [123]. We later proved that iNOS is a biomarker of poor prognosis and a good therapeutic target in a cohort of 73 TNBC patients [93]. In a previous report, we identified two genes, *RPL39* (ribosomal protein L39) and *MLF2* (myeloid leukemia factor 2), that are commonly mutated in lung metastases from breast cancer patients, and their inhibition significantly reduced BCSC self-renewal and number, tumor cell migration, invasion and generation of lung metastases, and tumor growth in in vitro and patient-derived xenografts (PDX) models of TNBC. Mechanistically, RPL39 and MLF2 expression was associated with iNOS signaling, and their mutations were associated with shorter median time to relapse in a cohort of 53 breast cancer patients, which suggests that iNOS inhibition represents a promising strategy for the treatment of TNBC [124]. In this regard, we reported that iNOS inhibitors diminish cancer cell proliferation and migration, CSC self-renewal and EMT by a targeting HIF1α and endoplasmic reticulum stress-transforming growth factor (TGFβ)-ATF4/ATF3 crosstalk [93]. Furthermore, we later confirmed that ATF4 is a transcriptional target of TGFβ-Smad2/3, is a biomarker of poor prognosis in TNBC patients, and promotes tumor progression by modulating CSCs, metastasis, relapse, and growth in PDX of TNBC [125]. Among the inhibitors tested, we reported that the pan-NOS inhibitor L-NMMA (NG-monomethyl-L-arginine) decreased cell proliferation, migration, and CSC self-renewal in vitro, and tumor growth (associated with less expression of Ki67), CSC self-renewal and tumor initiation in xenograft models of TNBC. Accordingly, we designed a safe and effective targeted therapy in TNBC by repurposing L-NMMA, previously studied in septic shock, with a dose regimen in combination with docetaxel that restrained tumor growth and prolonged mice survival [93]. Moreover, in combination with docetaxel, iNOS inhibition with L-NMMA enhanced the response to chemotherapy in PDX models of TNBC [94]. The translation of this therapeutic approach into clinic is under investigation in a phase Ib/II study in refractory locally advanced or metastatic TNBC patients (NCT02834403) [93,94]. Finally, iNOS has been associated with different signal transduction pathways such as vascular endothelial growth factor (VEGF). Increased levels of VEGF have been found in TNBC and it is known that NO can be responsible for it. Both iNOS and eNOS can induce VEGF and promote angiogenesis, thus L-NMMA (pan-NOS inhibitor) may be a good option to target this pathway [126].

### 4.5. Anexelekto (AXL)

AXL, named from the Greek word *anexelekto* which means “uncontrolled,” is one of the TAM (Tyro3, AXL, and Mer) family of receptors tyrosine kinase (RTK) [127]. Structurally, in the extracellular part, it is composed of two immunoglobulin-like domains and two fibronectin III domains. The intracellular part presents an RTK domain that contains a KWIAIES motif of TAM family. Its activation results in the autophosphorylation at the cytoplasmic domain that unleashes different cascades and downstream targets that are highly context dependent. Some of these pathways are PI3K/protein kinase B (Akt), extracellular-signal-regulated kinase (ERK), and STAT, which can stimulate tumorigenic processes such as cell motility, invasion, or proliferation [128]. In TNBC patients, the high expression of AXL is a predictor of poor prognosis, produces mesenquimal phenotypes, by promoting EMT through the expression of Vimentin, Twist, Snail, and Slug, higher chemoresistance, tumorigenesis, metastases, and CSCs, which make it a potential candidate to treat TNBC [80,128,129]. AXL can be activated by mechanisms dependent and independent of the ligand GAS6. If it is mediated by GAS6, AXL activates signaling pathways like PI3K/Akt, MAPK, NF-κB, and JAK/STAT, which can stimulate tumorigenic processes. On the other hand, the GAS6-independent pathway involves EGFR that activates AXL, which finally unleashes Akt transcription and produces an increase of tumor cell proliferation and migration [128]. Targeted inhibition of EGFR may not be a good option in TNBC because AXL can be activated thought other pathways and the response to EGFR inhibitors is limited [130]. Because of drug repositioning three drugs included in the same family are considered as a possible CSC-targeted therapy.

Phenotiazines. Goyette et al. carried out a research of drug repurposing based on AXL knockdown gene signature. Using CMap, they found that three phenothiazines (thioridazine, fluphenazine, trifluoperazine) could produce a similar gene signature. These dopamine receptor antagonists are used as anti-psychotics and were tested in TNBC, obtaining good results both in vitro and in vivo. In vitro, decrease of cell invasion, proliferation and viability, and increase of apoptosis were seen in TNBC cell lines. Interestingly, an increased sensitivity to standard chemotherapy was also observed in combination with paclitaxel. In vivo, a significant reduction of tumor growth and metastasis were observed. Furthermore, mechanistic insights revealed that these compounds did not exert their activities by antagonizing with dopamine receptor. AXL activity was not decreased but a reduction of PI3K/Akt/mammalian target of rapamycin (mTOR) and ERK signaling was produced, unravelling that repurposed drugs generate the same consequences as AXL knockdown [80].

## 5. Drug Repositioning to Target Cancer Stem Cells in TNBC

The CSC model for tumor propagation underlines that solid tumors are hierarchically organized, and contain a subset of cancer cells with stem-cell-like characteristics known as CSCs or tumor-initiating cells, which are able to sustain tumor growth, progression, and recurrence, as well as metastasis. Consequently, this model would explain intra-tumor heterogeneity and dormant behavior of several types of cancer [131,132,133]. CSCs phenotype varies according to the type of cancer. BCSC are characterized by surface markers CD44^+^/CD24^–/low^ and aldehyde dehydrogenase 1 (ALDH1) enzyme activity. Interestingly, it has been suggested that the acquisition of a stemness phenotype in CD44^+^/CD24^–/low^ subpopulation is connected to EMT [134], key event in metastatic spread [131,135,136]. EMT is known to be regulated by different pathways, including the TGFβ, PI3K/Akt/mTOR, MAPK, or Wnt/β-catenin, which can be abnormally regulated during malignant processes in TNBC [131]. In fact, several studies have demonstrated that activation of EMT induced by TGFβ increases the subpopulation of CSCs in breast cancers [137,138]. Interestingly, CSCs have been proved to be more abundant in TNBC than in other breast cancer subtypes, which could explain its higher aggressiveness [139,140]. Therefore, efforts are being focused on the development of CSC-targeted therapies [141]. Additionally, several studies have shown that CSCs are intrinsically resistant to chemotherapy and radiotherapy, therefore, targeting CSCs in combination with conventional chemotherapy might decrease the aggressiveness of TNBC and prevent cancer relapse and improve survival [131,132,133]. It has been suggested that EMT inhibitors could be potential CSC-targeted therapies in breast cancer. In fact, activation of Wnt/β-catenin signaling has been correlated with the expression of CD44^+^/CD24^–/low^ CSC subpopulation. Whereas different Wnt inhibitors are currently under development for the treatment of cancer, several FDA-approved drugs, such as salinomycin, vitamin D3, or pyrvinium pamoate, have proven to inhibit this pathway, being possible candidates for repurposing [50,142]. Some other FDA-approved drugs have also been demonstrated to regulate EMT and/or affect CSCs via different molecular pathways, such as all-trans retinoic acid (ATRA) [143], benztropine mesylate [144,145], and chloroquine [146]. Moreover, some of the previously mentioned TNBC-directed repurposed drugs were shown to target EMT or CSCs as well, including flubendazole, niclosamide, zoledronic acid, and L-NMMA. All breast CSCs-targeted drugs that are being investigated are summarized in Table 5. 

Salinomycin. It has been shown that LRP6, a co-receptor in the Wnt/β-catenin signaling pathway, is upregulated in TNBC, [158], and transcriptional knockdown decreased Wnt/β-catenin signaling, suppressing tumor growth in vivo [159]. Interestingly, the antibiotic salinomycin was demonstrated to induced the degradation of LRP6, inhibiting the Wnt pathway [147]. Gupta et al. studied the effects of salinomycin both in vitro and in vivo in comparison with paclitaxel. Salinomycin was found to decrease CD44^+^/CD24^−/low^ population both in cell culture and tumorspheres, whereas paclitaxel induced an increase of this cell population, showing that CSCs were resistant to paclitaxel but sensitive to salinomycin. This effect was later confirmed in mice orthotopically injected with SUM159 cells; it was shown that, compared to paclitaxel, salinomycin was able to inhibit tumor growth and the expression of CSC genes [149]. Moreover, a study investigating the efficacy of salinomycin in combination with LBH589 was proven to be a potential BCSCs-targeted therapy in TNBC by inducing apoptosis, arresting the cell cycle, and regulating EMT in breast CSCs [148].

Pyrvinium pamoate. This FDA-approved anthelmintic was discovered to inhibit the Wnt/β-catenin signaling pathway using a high-throughput screen in a *Xeropus* egg extract [160]. As a consequence of this inhibition, this drug is able to suppress self-renewal of CSC, it reduces both CD44^+^/CD24^−/low^ and ALDH^+^ BCSCs and expression of EMT markers such as N-cadherin, vimentin, and Snail [142]. Furthermore, pyrvinium pamoate inhibits PI3K-dependent pathway via suppression of Akt/P70S6K signaling axis [151], as well as mitochondrial respiration function [161] and fatty acids and cholesterol anabolism, lipids that are crucial to Wnt/β-catenin pathways [150]. Reduction of tumor growth was observed in in vivo assays [142,151]. Xu et al. suggested that pyrvinium pamoate’s effect on chemoresistance should be assessed in combination with traditional treatments based on the known association between BCSCs and Wnt pathways and the development of drug resistance [142].

Vitamin D3. Upon binding to its ligand, the vitamin D3 nuclear receptor (VDR) heterodimerizes with the retinoid X receptors (RXRs) and regulates the transcription of several genes involved in Wnt, TGFβ and Notch pathways in different types of cancer [143]. In breast cancer, vitamin D3 has been proved to decrease transcriptional levels of the Notch ligands, resulting in the inhibition of Notch-1 signaling, and levels of NF-κB1 [152,153]. Moreover, vitamin D3 has been shown to induce the downregulation of BRCA-1 expression, a commonly mutated gene in breast cancer, including TNBC [162]. In addition, Vitamin D3 was shown to reduce cell proliferation, CD44^+^/CD24^−/low^ population, and mammosphere formation [153]. Interestingly, Pervin et al. reported that, in breast cancer, VDR silencing was associated with EMT and a higher ability to form mammospheres, whereas its over-expression was followed by a decrease in mammosphere-forming ability. Moreover, in accordance with the inherent aggressiveness of TNBC, they reported that VDR was significantly downregulated in TNBC cells, which resulted in a relative insensitivity to vitamin D3 treatment. Accordingly, these authors showed that a combination therapy with DETA NONOate achieved a significant decrease in mammosphere formation in vitro and tumor growth in vivo [154]. Accordingly, vitamin D3 has been suggested to be a potential inhibitor of breast CSCs.

All-trans retinoic acid (ATRA). Also called tretinoin, is a retinoid used in dermatology which was approved to treat acute promyelocytic leukemia and has been investigated for the treatment of other cancers like lymphoma, leukemia, melanoma, lung cancer, or cervix [143]. In a HER2+ breast cancer cell line, Zanetti et al. proved that treatment of both ATRA and EGF suppressed tumorigenic effects of EGF. While EGF-treated cells developed an increase of Notch1 transcription and TGFβ pathway stimulation via SMAD3, ATRA+EGF-treated cells did not enhance levels of Notch1, and SMAD3 active form was also decreased as phosphorylation did not ensue. Hence, ATRA modulated and reduced EMT by inhibiting transcription of Notch1 and switching TGFβ pathway from a pro-migratory to anti-migratory program. In TNBC, further studies are needed to be done to verify these mechanisms [163]. Using CMap and introducing six analyses of up and down-regulated genes related to CSCs, Bhat-Nakshatri et al. found ATRA to be a good candidate for a CSC targeted therapy in breast cancer, although its effectiveness depends on tumor type. These gene signatures were obtained by comparison of gene expression in two opposite contexts: one associated with CSC versus a non-CSC conditioned control. In TNBC, it was more interesting in those subtypes having mesenchymal properties, as they are enriched for CD44^+^/CD24^–/low^ subpopulations. In vitro, ATRA produced a decrease in CSC self-renewal, determined by a mammosphere assay, and its effectiveness was augmented in cell lines with higher SOX2 expression. In addition, ATRA reduced levels of EGFR, SERPINE1, and Slug in a cell-line-type-dependent manner. MDA-MB-231 cell line was less sensitive to ATRA because of SOX2-independent characterization and KRAS mutation, which was responsible for resistance to ATRA. Thus, better results in mammosphere assays were obtained after the inhibition of KRAS pathway [155]. Furthermore, Ginestier et al. proved that treatment of ATRA reduced breast ALDH1^+^ CSC population [156]. 

Benztropine mesylate. It is used for the treatment of Parkinson’s disease. It acts as a central anticholinergic agent, as well as an antihistamine and a dopamine re-uptake inhibitor. Cell-based phenotypic screening and functional assays showed that benztropine mesylate inhibited mammosphere formation and self-renewal, reduced CSC subpopulations (both ALDH1^+^ and CD44^+^/CD24^–/low^), and improved chemotherapy in vitro. In vivo, it impaired CSC frequency and their tumor-initiating potential [144]. In addition, Sogawa et al. studied that benztropine could modulate EMT via STAT3, NF-κβ, and β-catenin in colorectal cancer [145].

Chloroquine. It is an autophagy inhibitor primarily used as an antimalarial drug. Interestingly, autophagy has been associated with drug resistance and maintenance of CSC population. In accordance with this mechanism, Choi et al. identified chloroquine as a potential repurposed BCSC inhibitor after in silico gene expression signature analysis of CD44^+^/CD24^−/low^ population. In vitro assays showed that chloroquine alone reduced the mammosphere formation efficiency and CD44^+^/CD24^−/low^ population in SUM159 and MDA-MB-231 cells, which was associated with a decrease in the expression of Jak2 and DNA methyltransferase 1 (DNMT1). Moreover, chloroquine sensitized TNBC cells to paclitaxel through the inhibition of autophagy. In vivo assays with an orthotopic xenograft model proved that chloroquine plus paclitaxel significantly reduced tumor growth and CD44^+^/CD24^−/low^ population, as opposed to paclitaxel alone, which had no effect on tumor growth and increased the CD44^+^/CD24^−/low^ population, compared to controls, in accordance with previous in vitro assays [146]. A phase II clinical trial demonstrated the efficacy of chloroquine in combination with taxanes in the treatment of patients with advanced or metastatic anthracycline-refractory breast cancer (NCT01446016). Among their results, objective response rate (ORR) was 45.16%, patients showed a median PFS of 12.4 months and a median OS of 25.4 months. The combination was well tolerated, with only up to 13.15% of patients experiencing Grade ≥ 3 adverse events. These results suggest that chloroquine, in combination with taxanes, could be used for the treatment of TNBC patients [157].

**Figure 4:**
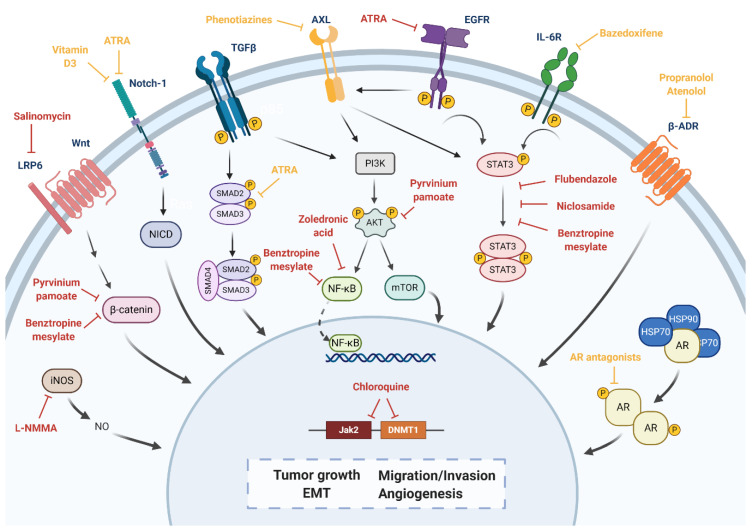
Overview of the different pathways investigated by drug repurposing to target breast cancer stem cells (BCSCs) and their potential inhibitors/modulators. Repurposed inhibitors under investigation are shown in red. Hypothesized inhibitors are shown in yellow. Created with BioRender.com.

Several of the previously mentioned target pathways in TNBC have been associated with EMT mechanisms, maintenance of tumor-initiating cells and/or tumor invasion, and drug resistance, including AR, ADR, STAT3, and AXL pathways. Correspondingly, we hypothesize that AR antagonists [56,58], the β-blocker propranolol [65] and atenolol [66,67,111], the STAT3 inhibitor bazedoxifene [71,72,118] and zoledronic acid [91], and phenothiazines (thioridazine, fluphenazine, trifluoperazine) [80] could act as potential inhibitors of BCSCs. Nevertheless, further investigations would still need to be performed. The pathways altered by these drug candidates to be potentially repurposed, as well as those included in Table 5, have been summarized in Figure 4.

## 6. Conclusions

The absence of targeted therapies for the treatment of TNBC, besides its inherent molecular and histopathologic complexity, strongly reduces the chance of patient recovery and life expectancy. It has therefore become imperative to find effective molecularly targeted treatments to overcome the aggressive progression of this breast cancer subtype. Whereas de novo research is a costly and long-term process, drug repurposing provides the possibility to reduce the time and investment needed to translate a drug from bench to bedside for a specific therapeutic purpose. Drug repositioning is achieved by means of different strategies, especially those including computational methods. Accordingly, several therapies with different molecular targets are currently being investigated for repurposing in TNBC, including androgen receptor, adrenergic receptor, STAT3, nitric oxide synthase, or AXL-directed therapies. However, because of the importance of CSCs in the progression and aggressiveness of this subtype of cancer, current efforts are also being directed to the search of compounds targeting this subset of tumor-initiating cells in TNBC. Herein, according to all repurposed drugs that are currently being studied for the treatment of TNBC, a few of them can be highlighted. AR antagonists bicalutamide, enzalutamide, and seviteronel, currently under clinical trials, seem to be particularly promising drugs in light of their association with the Wnt pathway, reduction of drug resistance, and induction of radiosensitization, respectively. However, clinical trials are evaluating the efficacy of these antiandrogens only in patients with a LAR subtype and, as a consequence, these drugs might not be successful in treating the rest TNBC patients. Other drugs that are currently in the clinical stage are also highlighted, including zoledronic acid, L-NMMA, and chloroquine. They decrease tumor viability, reduce CSC population and their capacity of self-renewal both in vitro and in vivo. Furthermore, they seem to sensitize these cells to chemotherapeutics, hence diminishing drug resistance. Finally, there are other drugs at preclinical stage that must be highlighted because they target CSCs or have been associated with a reduction of drug resistance, such as salinomycin, pyrvinium, vitamin D3, ATRA, benztropine, flubendazole, niclosamide, or propanolol. These drugs could be used as a monotherapy or in combination with chemotherapy to enhance the therapeutic response.

At the core of precision oncology, the high heterogeneity and molecular subtypes of TNBC should drive the diversity of approaches to tackle it, however, most studies do not discriminate between different subtypes. To date, only LAR subtype has really been addressed as an example of successful personalized drug repurposing. Besides the variety of molecular targets, a plethora of computational strategies hinder the ability to efficiently find potential repurposed drugs for TNBC patients. While having different tools for drug repositioning offers indeed a wide range of possibilities for personalized medicine, lack of a standardized protocol and a resolution of the most effective approach in the search of new uses for old drugs, raises the question: can computational drug repurposing actually be implemented as an improved method for drug discovery in personalized medicine and, more particularly, for TNBC? Factually, it is noticeable that some of the reviewed studies date from some years ago but none of those repurposed compounds have been yet approved for TNBC. While drug repurposing might increase the chances to help find new molecularly targeted candidates, hence improving the development of a more personalized medicine, the results suggest that not all candidates were as adequate as they might have seemed during in silico analysis, meaning that computational drug repurposing could not be as efficient as expected. It is therefore necessary for computational approaches to be validated and standardized, so as to reduce the chances of failure and allow drug repurposing to become an improved and attainable alternative with guarantees for personalized medicine. Be that as it may, drug repositioning has allowed to find new candidates that would not have been considered otherwise, making it still a powerful alternative for the search of a personalized treatment for TNBC patients. 

## Figures and Tables

**Table 1:** Summarized approved agents for non-metastatic triple-negative breast cancer (TNBC).

Class	Agent	Mechanism	Original Indication
Microtubule inhibitors	Paclitaxel Docetaxel	Disruption of microtubule dynamics leading to the end of cell division.	Ovarian cancer, atrial restenosis hormone-refractory prostate cancer
Anthracyclines	Doxorubicin, Epirubicin	Inhibition of DNA, RNA synthesis forming an anthracycline-DNA-topoisomerase II ternary complex. Harm of mitochondrial function. Generation of oxygen-free radicals. Activation of apoptosis and matrix metalloproteinase. Immune reactions.	Antibiotics from *Streptomyces peucetius* bacterium
Alkylating agents	Cyclophosphamide	Inhibition of DNA replication.	Immuno-modulator in autoimmune diseases. Immunosuppressant
Antimetabolites	Methotrexate	Antagonist of dihydrofolate reductase. Decrease the synthesis of purines and pyrimidines.	Leukemia
	Capecitabine	5-fluorouracil pro-drug. Inhibition of thymidylate synthetase.	Colon cancer
	Gemcitabine	Analogue of cytidine. Irreparable errors that inhibit DNA replication.	Anti-viral drug
Platinum	Carboplatin, Cisplatin	Damage of genetic material	Testicular, ovarian, and bladder cancers

**Table 2:** Novel approved agents for metastatic TNBC.

Class	Agent	Mechanism	Original Indication
PARP inhibitors	Olaparib Talazoparib	Inhibition of PARP. Cell death due to accumulation of irreparable DNA damage.	Ovarian cancer with *BRCA* mutation
PD-L1 inhibitor	Atezolizumab	Block interaction with receptors PD-1 and reverse T-cell suppression.	Non-small cell lung cancer Bladder cancer
ADC	Sacituzumab govitecan	Targeted to Trop-2 and conjugated with SN-38, a DNA damaging agent.	-

**Table 3:** Summarized repurposed drugs to treat TNBC that are under investigation in the preclinical phase.

Mechanism	Compound	Pre-Clinical Effects	Original Indication	Repurposing Method	References
α-ADR antagonist	α -yohimbine	Reduction of tumor growth in vitro. Development of resistance to paclitaxel when treated in combination with catecholamines and/or cortisol in vitro. Reversion of tumor growth after stimulation with clonidine in vivo.	Impotence	Non computational: target-based	[59,60,61]
Non-selective β1/β2-blocker	Propranolol	Inhibition of cell proliferation, arrest of the cell cycle at G0/G1 and S, and induction of cell apoptosis in vitro. Inhibition of tumor growth in vivo. Combination of propranolol with paclitaxel increased the anti-tumor efficacy of paclitaxel in vivo. Associated with less advanced disease at diagnosis and decreased risk of metastasis and mortality. Reverted isoproterenol-induced cell inhibition.	Hypertension	Non computational: target-based	[61,62,63,64,65]
Selective β1-blocker	Atenolol	Reduction of norepinephrine-induced cell migration in vitro. Inhibition of cell proliferation in vitro. Combination with metformin enhanced reduction of angiogenesis and metastasis in vivo. Not associated with differences tumor incidence, risk of metastasis and mortality rates. Associated with significantly lower recurrence but no significant OS.	Hypertension	Non computational: target-based	[63,66,67,68,69,70]
	Metoprolol	Associated with significantly lower recurrence but no significant OS.	Hypertension	Non computational: target-based	[68]
STAT3 inhibitor	Bazedoxifene	Decrease of cell viability, migration, colony formation. Increase cell apoptosis. Improvement of sensitivity to paclitaxel if combination.	Osteoporosis	Computational: structure-based	[71,72]
	Flubendazole	Inhibition of cell proliferation in vitro and tumor growth in vivo. Reduction of CD44^high^/CD24^low^ CSC population, mammosphere-forming ability and expression of stemness genes. Improvement of sensitivity to fluorouracil and doxorubicin if combination.	Anthelmintic	Non computational: target-based	[73]
	Niclosamide	Inhibition of cell proliferation in vitro and tumor growth in vivo. Reversion of EMT and inhibition of stem-like phenotype in cancer cells. Radiosensitizer in vitro and in vivo.	Anthelmintic	Non computational: screening	[74,75,76]
	Osthole	Induction of apoptosis in vitro. Reduction of tumor growth in vivo.	Osteoporosis	Non computational: literature-based	[77,78]
	Risedronate Sodium	Toxicity in TNBC cells in vitro.	Osteoporosis	Computational: structure-based	[79]
AXL pathway modulator	Thioridazine Fluphenazine Trifluoperazine	Decrease of cell invasion, proliferation, and viability and increase of apoptosis in vitro. Reduction of tumor growth and metastasis in vivo.	Anti-psychotics	Computational: transcriptional signature-based	[80]

**Table 4:** Summarized repurposed drugs for TNBC under current investigation in clinical trials.

Mechanism	Compound	Preclinical and Clinical Effects	Clinical Trials ^1^	Original Indication	Repurposing Method	References
AR antagonist	Bicalutamide	Reduction of cellular proliferation and colony formation, and induction cell apoptosis in vitro. Decreased cellular viability and induced apoptosis in vivo. CBR at 6 months of 19% and median PFS of 12 weeks (*n* = 26; AR expression higher than 10% by IHC). Grade 1–3 AEs included fatigue, limb edema, or hot flashes.	Phase II—completed (NCT00468715) Phase II—recruiting (NCT02605486) Phase III—recruiting (NCT03055312)	Prostate cancer	Non computational: target-based	[81,82]
	Enzalutamide	Reduction of cell proliferation, migration and invasion and increased apoptosis in vitro. Inhibition of tumor viability by inducing cell apoptosis in vivo. CBR at 16 weeks of 25%, median PFS of 2.9 months and median OS of 12.7 months (*n* = 118; AR expression higher than 0% by IHC). CBR at 16 weeks of 33%, median PFS of 3.3 months and median OS of 17.6 months (*n* = 78; AR expression higher than 10% by IHC). Grade 3 AEs included fatigue.	Phase II—completed (NCT01889238) Phase II—recruiting (NCT02689427) Phase Ib/II—active (NCT02457910)	Prostate cancer	Non computational: target-based	[55,56,83,84]
	Abiraterone acetate	Combination treatment with Chk1 inhibitors had an additive effect inhibiting cell apoptosis in vitro. Reduction of tumor growth, which was significantly higher with the combination treatment. CBR at 6 months of 20% and median PFS of 2.8 months (*n* = 30; AR expression higher than 10% by IHC). Grade 1/2 AEs included hypertension, fatigue, nausea, and hypokalemia.	Phase II—completed (NCT01842321)	Prostate cancer	Non computational: target-based	[85,86]
	Orteronel	Currently being investigated.	Phase II—active (NCT01990209)	Prostate cancer	Non computational: target-based	NCT01990209
	Seviteronel	Inhibition of cellular growth in vitro. Inhibition of tumor volume in vivo. Induction of radiosensitization, both in vitro and in vivo. Early results: CBR at 16 weeks of 33% (*n* = 6). Grade 1/2 AEs included fatigue, nausea and decreased appetite.	Phase I/II—completed (NCT02580448) Phase II—completed (NCT02130700)	Prostate cancer	Non computational: target-based	[87,88,89]
	Enobosarm	Currently being investigated.	Phase II—terminated (NCT02368691)	Prostate cancer	Non computational: target-based	NCT02368691
STAT3 inhibitor	Zoledronic acid	Induction of cell cycle arrest, decrease of cell viability, cell proliferation, self-renewal and expression of EMT markers in vitro. Antitumor potential with doxorubicin in vivo. Improvement of pCR and DFS in combination with chemotherapy versus only chemotherapy.	Phase II—completed (UMIN000003261) Phase II—terminated (low accrual rate) (NCT02347163) Phase II—recruiting (NCT03358017) Phase III—active (NCT02595138) Phase unknown—recruiting (NCT04045522)	Osteoporosis	Computational: structure-based, Non computational: literature-based	[79,90,91,92]
NOS inhibitor	L-NMMA	Decrease of cell proliferation, migration, and CSC self-renewal in vitro. Decrease of growth, CSC self-renewal and tumor initiation in xenograft models of TNBC. Improvement of chemotherapy response in combination with docetaxel in PDX models of TNBC.	Phase Ib/II—recruiting (NCT02834403)	Septic shock	Non computational: target-based	[93,94]

^1^ Last access to ClinicalTrials.gov on October 16th, 2020.

**Table 5:** Summary of drug candidates to target cancer stem cells (CSCs) under investigation by drug repurposing.

Mechanism	Compound	Cellular and Molecular Effects	Original Indication	Repurposing Method	References
Wnt, LRP6	Salinomycin	Decreased CD44^+^/CD24^−/low^ population both in vitro and in vivo. Inhibition of tumor growth and expression of CSC genes in vivo. Combination with LBH589 induced apoptosis and cell cycle arrest and regulates EMT in BCSCs.	Antibiotic	Non computational: high-throughput screening	[147,148,149]
Wnt/β-catenin, PI3K dependent pathway, lipid anabolism	Pyrvinium pamoate	Reduction of CSC self-renewal. Reduction of CD44^+^/CD24^−/low^ and ALDH+ populations. Reduction of expression of EMT markers (N-cadherin, Vimentin and Snail). Reduction of tumor growth in vivo.	Anthelmintic	Non computational: high-throughput screening	[142,150,151]
Notch-1, NF-κB1	Vitamin D3	Reduction of cell proliferation, CD44^+^/CD24^−/low^ population and mammosphere formation in vitro. Relative insensitivity to vitamin D3 treatment, but combination therapy with DETA NONOate achieved a significant decrease in mammosphere formation in vitro and tumor growth in vivo.	Vitamin supplement	Non computational: target-based	[152,153,154]
Notch-1, TGF-β	ATRA	Inhibition of mammospheres formation and reduction of CSC self-renewal. Reduction of ALDH1 CSC subpopulation.	Dermatologic diseases, acute promyelocytic leukemia	Computational: transcriptional signature-based	[155,156]
STAT3, NF-κB, and β-catenin	Benztropine mesylate	Inhibition of mammospheres formation and reduction of CSC self-renewal. Reduction of ALDH and CD44^+^/CD24^−/low^ populations.	Parkinson’s disease	Computational: cell-based phenotypic screening	[144]
Jak2, DNMT1	Chloroquine	Inhibition of autophagy. Reduction of mammosphere formation efficiency and CD44^+^/CD24^−/low^ population in vitro. Sensitization to paclitaxel through the inhibition of autophagy in vitro. Combination of paclitaxel significantly reduced tumor growth and CD44^+^/CD24^−/low^ population in vivo. Phase II clinical trial for chloroquine in combination with taxanes: ORR of 45.16%, median PFS of 12.4 months and median OS of 25.4 months. 13.15% of patients experienced Grade ≥ 3 adverse events.	Antimalarial	Computational: transcriptional signature-based	[146,157] NCT01446016
STAT3	Flubendazole	Loss of CD44^+^/CD24^−/low^ population. Decrease of mammosphere-forming ability. Suppression of stem cell genes expression.	Anthelmintic	Non computational: target-based	[73]
	Niclosamide	Reversion of EMT. Inhibition of stem-like phenotype.	Anthelmintic	Non computational: high-throughput screening	[74]
STAT3, NF- κB	Zoledronic acid	Induction of cell cycle arrest, decrease of cell viability, cell proliferation, self-renewal and expression of EMT markers in vitro.	Osteoporosis	Computational: structure-based. Non computational: literature-based	[91]
iNOS	L-NMMA	Decrease of mammosphere-forming ability.	Septic shock	Non computational: target-based	[93]

## Abbreviations

ADC	Antibody drug conjugate
ADR	Adrenergic receptor
AE	Adverse events
Akt	Protein kinase B
ALDH1	Aldehyde dehydrogenase 1
AR	Androgen receptor
AREG	Amphiregulin
ATRA	All-trans retinoic acid
AXL	Anexelekto
BARK	β-adrenergic receptor kinase
BCSC	Breast cancer stem cells
BL1	Basal-like 1
BL2	Basal-like 2
cAMP	Cyclic AMP
CBN	Causal biological networks
CBR	Clinical benefit rate
CMap	Connectivity Map
CPIs	Checkpoint inhibitors
CSC	Cancer stem cells
CTLA-4	Cytotoxic T-lymphocyte-associated antigen-4
DFS	Disease free survival
DTI	Drug-target interaction
EGF	Epidermal growth factor
EGFR	Epidermal growth factor receptor
EMT	Epithelial-to-mesenchymal transition
ER	Estrogen receptor
FAK	Focal adhesion kinase
FDA	Food and Drug Administration
FGF	Fibroblast growth factor
GEO	Gene Expression Omnibus
GPCR	G protein-coupled receptor
HER2	Human epidermal growth factor receptor 2
IGF	Insulin-like growth factor
IHC	Immunohistochemistry
IL-10	Interleukin 10
IL-6	Interleukin 6
IM	Immunomodulatory
ITT	Intent-to-treat population
JAKs	Janus kinases
LAR	Luminal androgen receptor
L-NMMA	NG-monomethyl-L-arginine
M	Mesenchymal
MAPK	Mitogen-activated protein kinase
mCRPC	Metastatic castration-resistant prostate cancer
MLF2	Myeloid leukemia factor 2
MSL	Mesenchymal stem–like
mTOR	Mammalian target of rapamycin
NO	Nitric oxide
NOS	Nitric oxide synthase
NOS1/nNOS	Neuronal nitric oxide synthase
NOS2/iNOS	Inducible nitric oxide synthase
NOS3/eNOS	Endothelial nitric oxide synthase
OS	Overall survival
PARP	Poly[adenosine diphosphate-ribose] polymerase
PDB	Protein Data Bank
pCR	Pathologic complete response
PD-1	Programmed cell death 1
PD-L1	Programmed cell death-ligand 1
PDX	Patient-derived xenografts
PFS	Progression free survival
PI3K	Phosphatidylinositol-3 kinase
PKA	Protein kinase A
PPI	Protein-protein interaction
PR	Progesterone receptor
PRISM	Protein Interactions by Structural Matching
ROS	Reactive oxygen species
RPL39	Ribosomal protein L39
RTK	Receptors tyrosine kinase
RXR	Retinoid X receptors
SARMs	Selective androgen receptor modulators
SAEs	Serious adverse events
STAT3	Signal transducer and activator of transcription 3
TAM	Tyro3, AXL and Mer
TCGA	The Cancer Genome Atlas
TGFβ	Transforming growth factor β
TILs	Tumor infiltrating lymphocytes
TNBC	Triple-negative breast cancer
Trop-2	Trophoblast cell-surface antigen 2
VDR	Vitamin D3 nuclear receptor
VEGF	Vascular endothelial growth factor
VHTS	Virtual high-throughput screening
